# Dogs as a model to study the emergence of concept manipulation skills for language-readiness

**DOI:** 10.1007/s42977-025-00267-1

**Published:** 2025-06-25

**Authors:** Dorottya S. Rácz, Marianna Boros, Attila Andics

**Affiliations:** 1 Neuroethology of Communication Lab, Department of Ethology, Eötvös Loránd University, Budapest, Hungary; 2 Doctoral School of Biology, Institute of Biology, Eötvös Loránd University, Budapest, Hungary; 3 ELTE NAP Canine Brain Research Group, Budapest, Hungary

**Keywords:** Language evolution, Social-cognitive skills, Event perception, Symbolic capacities, Syntactic abilities, Dog

## Abstract

Language-readiness entails the ability to segment holistic events into discrete concepts, learn signals for such concepts, and combine them in a rule-based manner to create composite meanings. There is much debate about whether, and to what extent, the brain mechanisms that enable concept manipulation abilities in humans are unmatched in the animal kingdom. Challenging human-uniqueness theories, we propose a social cognition-mediation account hypothesizing that concept manipulation abilities essential for language-readiness could also emerge in other species with a sufficient level of certain prerequisite social-cognitive skills, namely goal-representation, intentionality-attribution, and mentalization. We argue for the involvement of a new species in comparative studies on language evolution to evaluate this hypothesis: the domestic dog, a species that has undergone selective pressures for prosociality during domestication similar to those experienced by early humans, as well as shows a natural propensity to communicate their experiences. As a consequence, dogs may possess the necessary social-cognitive capacities to develop concept manipulation skills. Dogs’ concept manipulation abilities have never been systematically investigated, nor directly compared to those of humans. Capitalizing on recent advances in comparative non-invasive neuroimaging and behavioural measures, here we propose feasible, promising experimental approaches for such investigations.

## The emergence of a language-ready brain

From the long list of the set of abilities that have been suggested by language evolution theorists to render a brain language-ready, the capacity for symbolic processing and the tracking of nested hierarchical structures are the most agreed upon (Dehaene et al. 2015; Fischmeister et al. 2017; Harnad 1990, 1996; Hauser et al. 2002). The former enables humans to efficiently form concept-label associations, while the latter gives rise to the complex human syntactic ability, allowing to form new, composite meanings from the rule-based combinations of label-mediated symbols. A long-standing debate concerns the origin of these abilities: are they the result of an abrupt change in the hominin line (Chomsky 1965; Pinker 1994) or did they evolve from shared foundations of social and cognitive abilities with gradual changes in one or multiple mechanisms (Fitch 2017). Challenging human uniqueness accounts, comparative efforts have mostly focussed on nonhuman primates, due to their phylogenetic proximity, and birds, due to their advanced vocal learning capacities; but evidence for concept manipulation abilities necessary for language-readiness remains weak, mostly limited to case studies on symbolic capacities (Ekström 2023; Gillespie-Lynch et al. 2014; Lyn and Savage-Rumbaugh 2000; Shanker et al. 1999) and less complex hierarchies (Corballis 2007; Fitch and Hauser 2004; ; Liao et al. 2022; Wilson et al. 2020). So, identifying which predispositions and through what mechanisms contributed to the emergence of language-readiness in early humans remains a crucial knowledge gap.

**Fig. 1:**
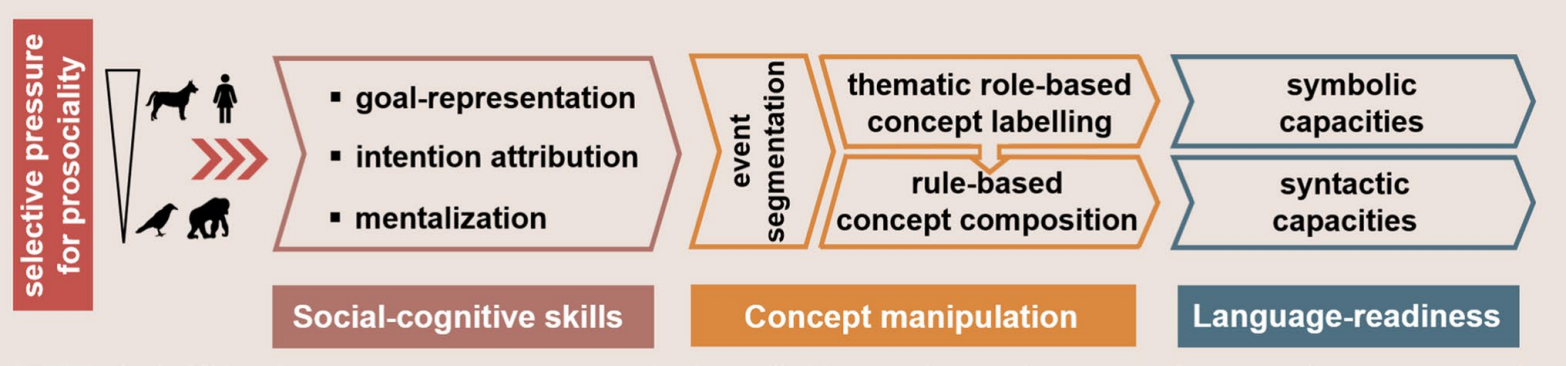
The social cognition-mediation theory of concept manipulation skills for language-readiness

Here, we propose a synthesis of linguistic and comparative theories of language evolution by putting forward a social cognition-mediated concept manipulation account of the emergence of language-readiness in the mammalian brain. Concept manipulation entails the efficient segmentation of events into discrete thematic roles (agents, actions, patients), which provides the basis of concepts in humans (Rissman and Majid 2019). These concepts can be associated with labels (forming symbols) and through their rule-based manipulation we arrive at complex syntax. Social cognition-mediation of concept manipulation refers to the hypothesis that the concept manipulation skills for language-readiness that are possessed by humans require complex social-cognitive abilities, such as goal-representation, intention attribution and mentalization (Fig. 1; Strickland et al. 2014; Zuberbühler and Bickel 2022). These prerequisite social skills have likely emerged in early humans due to the selective pressure of domestication and functional selection for cooperation which together promoted prosociality, in the form of e.g. attachment behaviour, sensitivity to communicative cues or reduced aggression (Hare 2017) Miklósi and Topál 2013). Nonhuman primates and birds, however, have never been subject to such selective forces. But there is a species that has undergone similar selective pressures as early humans and possesses complex social-cognitive skills: the domestic dog (*Canis familiaris*). In this paper, we will argue that involving dogs in comparative studies on language evolution can thus be an optimal approach to evaluate whether possessing complex social-cognitive capacities provides sufficient ground for concept manipulation skills to emerge.

### Event segmentation behind linguistic abilities

The human brain has a tendency of automatically segmenting continuous experience into discrete events consisting of thematic roles, namely agents, actions and patients, and subsequently build conceptual knowledge through repeated exposure and abstraction (Rissman and Majid 2019). Event segmentation experiments typically use a paradigm in which subjects watch videos of activities, and they are asked to press a button whenever they think a meaningful event ends. Results show that the boundaries are usually drawn between scenes when who-does-what-to-whom patterns change, and resulting chunks have their readily identifiable elements: an agent (e.g. a person or an animal), the entity performing an action (e.g. kicking, throwing, speaking); a patient, the entity (e.g. a person, an animal or an object) undergoing or affected by an action (Speer et al. 2007; Zacks et al. 2001a, 2001b). Segmenting events into these thematic roles is crucial for forming and labelling semantic categories, and thus for symbolic capacities to emerge. Also, humans understand events as having a temporal structure and containing dependencies across thematic roles and event sequences (Jackendoff 2002). Importantly, this mental structuring of events closely parallels syntactic parsing in language, which organizes sentences into subjects, verbs (predicates) and objects. Therefore, syntax in humans is argued to originate naturally from how humans perceive events (Zuberbühler 2019, 2020) and derives from the tendency of the human brain to detect who-does-what-to-whom patterns. Thus, how humans perceive events can provide the basis for the emergence of concepts, which can be associated with symbols (concept labelling) and manipulated in a rule-based manner (concept combination), to therefore arrive at semantic and syntactic abilities.

### Social-cognitive skills necessary for event segmentation

Representing social interactions in complex ways, attributing goal-directed behaviour and assigning intentionality to agents is argued to be crucial for segmenting events into thematic roles (Strickland et al. 2014; Wilson et al. 2022; Zuberbühler 2020; Zuberbühler and Bickel 2022). Humans do not merely perceive events as assemblies of categorical information, nor do they view agents and patients as simply two participants acting simultaneously and independently but rather, they detect the causal dependence between them. To represent an event as more than a chain of accidental actions and interactions between the protagonists, it is necessary to recognize different forms of causality and to think of others as intentional entities acting to achieve a goal. The attribution of intentions is also necessary to identify goals in cases where they are not visible or easy to detect, since they are derived from internal psychological states and motives rather than from physical causations (Wilson et al. 2022). Additionally, for efficient event segmentation, complex mentalization skills such as perspective taking (i.e. appreciating that others may not have the same perceptions, mental states or knowledge as we have) are also considered to be important, especially for understanding unexpected actions of an agent (Myowa-Yamakoshi et al. 2015).

Consistently, adult-like event perception is not present from birth but seems to develop gradually in parallel with these social-cognitive capacities (i.e. the representation of others’ goals, the attribution of intentionality and mentalization skills). Indeed, 6-month-olds and younger age groups cannot reliably anticipate action goals (Falck-Ytter et al. 2006; Kanakogi and Itakura 2011), do not seem to understand or care about others’ intentions (Woodward 2003), and do not show perspective taking skills (Ikeda et al. 2022; Moll and Tomasello 2006). Consistent with this, when watching short videos of natural events in which agents act on patients (e.g. someone is reading a book, grooming), young infants do not track event roles like adults: they tend to attend mostly to background information, not to action-patient relations, as measured by fixation preferences (Wilson et al. 2024). However, from about 7 months of age children show goal-directed eye movements when observing simple actions (e.g. reaching, putting) (Adam et al. 2020; Cannon and Woodward 2012; Falck-Ytter et al. 2006; Kanakogi and Itakura 2011; Woodward 1998). Children’s understanding that a sequence of actions can lead to an overarching goal (e.g. one’s goal was not only to reach the lid but to open it and grab an object) seems to develop by the end of the first year (Henderson and Woodward 2011; Woodward 1998). In addition, by 12 months, children attribute intentions to agents (Gergely et al. 1995; Phillips et al. 2002; Woodward 2003), for example they prefer individuals who display helping behaviour over hindering or neutral behaviour (Hamlin et al. 2007; Woo et al. 2024; Woo and Spelke 2023b) and, importantly, this choice depends on how the infant understands the goal of the agent (Woo and Spelke 2023a). Around one year of age, children also begin to represent others' (false) beliefs and evaluate their intentions according to what that person potentially knew (Woo and Spelke 2023b), as shown by looking time and choice preference measures, as well as they begin to understand that others' perspectives can be different from their own (Ikeda et al. 2022; Luo and Baillargeon 2007).

The second half of the first year is crucial in the development of event perception as well. In support, it has been shown recently that, although infants in their first year of life generally have coarser event structure, representing longer events with no evidence of timescale hierarchy, how well their neural representation of events matches that of adults correlates with age when measured with fMRI (Yates et al. 2022). In addition, although 6-month-olds do not yet prioritize agent-patient interactions when observing an event (Wilson et al. 2024), they already focus on agents and their goals when analysing an action looking longer to new goal than new path events, and they represent differently the action of a human and the motion of an object preferring the goal only when the agent is a hand, not an object (Woodward 1998). Also, 9-month-old children were shown to have better memory for events both when including a clear agent and when trained to perceive an inanimate object as an agent’s tool, suggesting their sensitivity to agent-patient relations (Howard and Woodward 2019). Moreover, pupillometry and habituation experiments showed that from about 8 months infants represent abstract agent and patient roles as they demonstrate surprise when roles eventually switch (Papeo et al. 2024), and show causal representations of events (Muentener and Carey 2010). Thus, children’s event segmentation capacity appears to develop as they gain a more complex understanding of the social world, further supporting the argument that these social skills are crucial for representing events as causally structured agent-patient interactions.

### Social-cognitive skills and concept manipulation capacities in nonhuman animals

Evidence for complex social-cognitive skills and concept manipulation capacities is mixed in nonhuman animals. Some studies suggest that nonhuman primates can interpret actions as goal-directed (Kano and Call 2014). Similarly, chimpanzees can infer the agent’s different goals in different contexts and act accordingly, even if the main actions are otherwise the same (Buttelmann et al. 2012). Nonhuman primates understand goal-directed actions with physical causality (e.g. if the barrier was removed, it should not be visible in the next scene), but only for actions that are familiar or within their own behavioural repertoire (Rochat et al. 2008; Wood et al. 2008). However, others argue that nonhuman primates may not understand goal-directed behaviours as humans do (Wilson et al. 2022). For example, although chimpanzees do understand goal-directed actions in which the goal is familiar and implicit, they seem to struggle with understanding actions with unexpected outcomes or that lack a specific target (Buttelmann et al. 2017; Myowa-Yamakoshi et al. 2015). This may be because in such events goal identification would require the understanding of others’ mental states and innate goals even when those do not meet the animal’s expectations. In other words, nonhuman primates detect goal-directedness in actions with simple physical causality but, unlike human adults, may struggle with psychological causality (Wilson et al. 2022). But despite such differences in goal representation and early reports that apes do not reliably distinguish intentional and accidental actions and show no clear preference for the former (Povinelli et al. 2007; Tomasello and Call 1997), more recent studies suggest that apes do have the capacity to attribute intention to others (Buttelmann et al. 2007, 2008); for review, see (Krupenye and Call 2019; Tomasello 2023). Finally, whether nonhuman animals exhibit perspective taking skills is also debated. Although evidence indicates that apes may be sensitive to the false beliefs of others, which per se requires perspective taking (Krupenye et al. 2016), other studies fail to show that nonhuman primates can access or care about the beliefs of others (for a review, see Krupenye and Call 2019).

The way nonhuman animals represent events remains largely unexplored. Experiments directly investigating this question are largely missing, and most existing (indirect) research has focused primarily on primate species. Evidence suggest that primates can attribute agency (Kano and Call 2014; Kupferberg et al. 2013; Myowa-Yamakoshi et al. 2012, 2015), discriminate agents and patients (McFarland et al. 2013; Myowa-Yamakoshi et al. 2012), as well as they seem to keep track of and detect causality in social interactions as measured by looking time preferences, gaze and vocal behaviour (Clay et al. 2016; Overduin-de Vries et al. 2016; von Rohr et al. 2015). However, it is unclear to what extent they decompose events into causally structured interactions between agents and patients. To date, only one study has directly compared event representation in apes and humans. The findings suggest that while apes, like humans, have an early focus on the agent-action-patient triad, they attend more to background information than humans when watching short videos (Wilson et al. 2024). In addition, apes engage less with agents throughout an event than humans (Myowa-Yamakoshi et al. 2015; Wilson et al. 2024), suggesting important species differences in event perception.

Nonhuman evidence for other concept manipulation skills is also controversial. Certain nonhuman animals (birds and primates) were shown to exhibit symbolic capacities, however, such reports are limited to case studies (Ekström, 2023; Gillespie-Lynch et al. 2014; Lyn and Savage-Rumbaugh 2000; Pepperberg 2002; Shanker et al. 1999). Similarly, some birds and certain primates potentially exhibit compositionality in their vocalizations, combining meaningful calls into sequences that elicit different behaviours (Arnold and Zuberbühler 2006; Ouattara et al. 2009; Spiess et al. 2024; Suzuki et al. 2016). Most sound sequences produced by animals, however, are just simple concatenations, combinations of units that are meaningless in themselves (Fishbein et al. 2020; Garland et al. 2017; Lameira et al. 2015). Also, gestures, facial expressions (for review, see Amici et al. 2024), and tool use (Putt et al. 2024) lack compositionality even in primates. Such differences between humans and nonhuman primates in some tendencies to represent events and other concept manipulation abilities may be related to differences in prerequisite higher-level social skills, which may only be partially shared by nonhuman primates.

### Selective pressures promoting social-cognitive skills and the emergence of a complex communicative system in humans

In early humans, the prerequisite social skills for concept manipulation have likely emerged due to selective pressures promoting prosociality. According to the self-domestication hypothesis, humans underwent similar evolutionary pressures as domestic animals, disfavouring aggression and favouring social tolerance (Hare 2017). These processes were probably triggered by different changes in the environment or community structures promoting sophisticated social-cognitive skills (Benítez-Burraco 2025; Brooks and Yamamoto 2021; Hare 2017; Pisor and Surbeck 2019; Raviv et al. 2023; Sánchez-Villagra and van Schaik 2019; Thomas and Kirby 2018). Similarly, when introducing the “human behaviour complex”, Csányi (2000) also argued that during early human evolution the expansion of social groups and the increased time spent together could only have emerged and been maintained with reduced intra-group aggression and the willingness to interact with others. Also, group cohesion required a high level of cooperation, sharing and helping behaviour (Csányi 2000), all of which rely on complex social skills. To efficiently help others or to cooperate with them, early humans had to be able to access what others wanted to achieve, requiring both the understanding of goal-directed actions (even complex, overarching goals) and attributing intentions to others (Warneken and Tomasello 2009). Perspective taking may also benefit cooperation, allowing individuals to better anticipate others' needs and intentions (Galantucci and Garrod 2011; Mouw et al. 2020; Novembre et al. 2019; Todd and Galinsky 2014).

Living in large, dense groups or even multilevel societies and facing everyday social challenges could thus create an environment for early humans in which gaining complex social-cognitive skills and developing a complex communicative system became beneficial. Specifically, increased social complexity and the willingness to cooperate likely required the formation of a decontextualized communicative system that would allow the interindividual synchronization of internal thoughts. Humans’ urge to communicate their experiences, thoughts and beliefs, and to refer to things that are not present, together could promote conceptual thinking, referentiality and abstraction (Csányi 2000), and lead to the emergence of language (Benítez-Burraco 2025; Benítez-Burraco and Kempe 2018; Cuskley and Sommer 2022). Thus, while these theories explain the contextual framework behind the emergence of language, the social-cognition mediation hypothesis of concept manipulation skills provides a mechanistic explanation of how social-cognitive skills could potentially lead to a language-ready brain through concept manipulation capacities.

However, the experimental verification of this hypothesis is still missing. The species which have been the focus of comparative studies on language evolution (primates and birds) have never been subject to similar selective pressures, and do not seem to exhibit an urge to communicate their experiences (Bullinger et al. 2014; Fischer 2021; Genty and Zuberbühler 2015). Therefore, here we propose a major paradigm shift, the involvement of domestic dogs in comparative studies on language evolution. Having been subjected to selective pressures similar to those experienced by early humans, dogs may possess the prerequisite social skills to serve as a suitable model species for testing the social cognition-mediation hypothesis behind the emergence of concept manipulation skills for language-readiness. Dogs’ propensity to communicate perceptions may also promote their linguistic abilities.

## Dogs as a model for evaluating the social cognition-mediation theory behind language readiness

Dogs from the beginning of their domestication history were immersed in the human social niche, facing similar social problems as humans to which they have had to adapt and modify their behaviour to succeed in the human environment (Hare and Tomasello 2005; Kubinyi et al. 2007; Miklósi 2007; Miklósi and Topál, 2013). In line with this, dogs spontaneously cooperate with humans (Naderi et al. 2001), and evidence also suggests that dog pups, unlike wolf pups, do not exhibit aggressive behaviour but show more communicative signals, preference (Gácsi et al. 2005) and even attachment behaviour to their caregivers (as evidenced in adult dogs: Gácsi et al. 2001; Topál et al. 1998). Additionally, dogs and wolves show remarkable differences in their sensitivity to human ostensive cues (Topál et al. 2009), and in initiating and maintaining communicative interaction with humans (Miklósi et al. 2003). Also, genetic distance from the wild ancestor affects dogs’ human-oriented social behaviours (Serpell and Duffy 2014; Smith et al. 2017) and their reaction towards communicative signals (barks and howls) (Lehoczki et al. 2023). Interestingly, recent findings suggest a molecular convergence underlying hypersocial behaviour in humans and dogs. Genetic variants linked to dogs’ human-directed sociability is associated with hypersociability in humans, pointing to potential shared biological foundations for heightened sociability across these species (Tandon et al. 2024).

There is ample evidence for dogs’ complex social-cognitive skills and sensitivity to even small indications of human communicative intent. Dogs understand human goal-directedness (Marshall-Pescini et al. 2014) and intentionality (Byrne et al. 2023; Heberlein et al. 2017; Horschler et al. 2022; Schünemann et al. 2021; Wallner Werneck Mendes et al. 2024). Moreover, after an interaction of a joint action with humans, dogs show a preference to re-engage with the former partner, which can be interpreted as a form of joint intentionality (mutual understanding of shared goals) (Byrne et al. 2023; Horschler et al. 2022). Dogs also appear to be able to differentiate which objects is relevant or irrelevant to a human partner and provide helpful information to find the desired one (Piotti and Kaminski 2016). In addition, in some situations dogs show spontaneous cooperative and helping behaviour towards humans when they are unable to solve a task alone (Bräuer et al. 2013; Csepregi and Gácsi 2023; Jaasma et al. 2020; Naderi et al. 2001). Such helping and cooperating behaviours provide further evidence that dogs may understand humans as intentional agents with goals. Furthermore, dogs are more likely to follow the instructions of a human who could see a food-hiding event than a human who could not (Catala et al. 2017). Also, similarly to children (Woo and Spelke 2023b), dogs show different responses when a human instructor/helper has true vs. false beliefs about the location of a hidden food, but both provide misleading information (Lonardo et al. 2021). The findings of these studies potentially suggest perspective taking in dogs and an understanding of what their partner is aware of. Additionally, while some studies suggest that apes communicate intentionally only in specific situations when their reproductive success is threatened (Genty and Zuberbühler 2015) and may not understand cooperative communicative intentions of humans (Herrmann and Tomasello 2006), dogs have a sophisticated ability to process human communicative cues as they engage in gaze following (Hare and Tomasello 2005), understand human pointing (Bray et al. 2021; Miklósi et al. 2005), and are sensitive to ostension (Byosiere et al. 2022; Tauzin et al. 2015). Dogs can also differentiate the focus of human attention (Virányi et al. 2004) and establish eye contact with humans (Bognár et al. 2021; Savalli et al. 2016; Topál et al. 1997). Most importantly, dogs exhibit an urge to communicate their experiences and engage in information sharing with humans (Piotti and Kaminski 2016) a key requirement for communicating referentially and intentionally. Indeed, dogs do not only understand but also spontaneously and efficiently use functionally referential communication during their social interactions with humans (Miklósi et al. 2000; Pérez Fraga et al. 2021, 2023; Savalli et al. 2014; Tauzin et al. 2015; Worsley and O’Hara 2018) more so than primates (Hare and Tomasello 2005; MacLean et al. 2017) or similarly kept pigs (Pérez Fraga et al. 2021, 2023) and cats (Miklósi et al. 2005), and from an early age (Gácsi et al. 2009; Riedel et al. 2008).

Altogether, while other nonhuman species may exhibit some of the social-cognitive skills required for concept manipulation as well, dogs appear to possess a more complete set of these prerequisites. These characteristics, together with their trainability and availability, place them in a better position than most other nonhuman species to develop concept manipulation skills, and thus to exhibit some syntactic and symbolic capacities. Therefore, we argue that dogs provide an ideal model species to evaluate the social cognition-mediation theory of the emergence of concept manipulation skills. The general prediction of this hypothesis is that dogs, being equipped with the necessary social-cognitive skills and with a propensity to communicate perceptions, are capable of efficient event segmentation and, in turn, may be well prepared to develop abilities for acquiring symbolic capacities and understanding syntax. On the contrary, if dogs turned out to exhibit systematic differences in certain concept manipulation skills of humans, while challenging the social cognition-mediation hypothesis of the emergence of concept manipulation skills, would be instrumental for identifying the mechanisms that after all helped humans to acquire superior symbolic and semantic capacities.

Additionally, dogs have been immersed in the human social-linguistic niche for thousands of years and on a daily basis (Larson et al. 2012; Miklósi 2007; Pongrácz et al. 2001; Thalmann et al. 2013). Therefore, speech is part of their everyday life, familiarizing them with a communication system characterized by complex statistical regularities, hierarchies and compositionality. Thus, in addition to the selective pressures acting on dogs, the socio-linguistic environment in which they live may also help them discover certain linguistic regularities and develop skills that enable syntactic capacities. These facts, together with their trainability and willingness to cooperate with humans, make dogs ideal subjects for comparative non-invasive neuroimaging linguistic experiments aimed at exploring what makes a brain language-ready.

### Event representation and linguistic abilities of dogs

Dogs’ event understanding has never been systematically investigated, but specific capacities relevant for their event perception have recently been described. Dogs, similarly to humans, discriminate between causal (i.e. chase) and independent motion patterns (Abdai et al. 2017), and readily perceive animacy (Abdai 2025; Abdai et al. 2022; Völter and Huber 2022), which is often a prerequisite for agency attribution (Jahan et al. 2018; Kittilä et al. 2011). Moreover, recent comparative fMRI experiments revealed that dogs’ mid suprasylvian gyrus is functionally analogous with agent-responsive brain regions in humans (Boch et al. 2024; Farkas et al. 2024) and that the dog brain has distinct representations of actions of animate entities (Phillips et al. 2022). Thus, it is possible that, despite their phylogenetic distance, dogs also evolved human-analogue event segmentation mechanisms, however, it is yet to be investigated.

Recent work, partly from our laboratory, also provided evidence for dogs’ various linguistic abilities. Similarly to humans, dogs track transitional probabilities to segment continuous speech (Boros et al. 2021). Dog brains can also discriminate a familiar language from an unfamiliar one, suggesting that they keep track of the auditory regularities that characterize languages (Cuaya et al. 2022). Although most dogs seem to be limited in their capacity to learn a large number of labels (Fugazza et al. 2021) a handful of dogs (over 40 individuals described globally) are capable of spontaneously and rapidly acquiring exceptionally large object-label vocabularies (of over 100 words) during everyday social interactions (Dror et al. 2021; Fugazza et al. 2021; Fugazza et al. 2021a, b). Also, typical dogs are estimated to respond to an average of 89 words—a number that should be treated with caution, as it comes from owner reports—suggesting that label learning is a natural part of their everyday lives (Reeve and Jacques 2022). In addition, besides dogs’ general capability to comprehend referential communication (Miklósi et al. 2000; Pérez Fraga et al. 2021, 2023; Savalli et al. 2014; Tauzin et al. 2015), our recent discovery of semantic expectation violation ERPs for mismatched object-label associations suggests that even typical dogs have referential understanding of object words (Boros et al. 2024). It has also been evidenced that dogs can process word meanings without any gestural or intonational cues (Andics et al. 2016; Gábor et al. 2020). Moreover, case studies indicate that some dogs also have at least limited compositional abilities, comprehending two-item sentences (Pilley and Reid 2011; Ramos and Ades 2012). But the complexity and the generality of this meaning composition capacity in dogs have never been investigated. Also, whether dogs can track hierarchically nested structures, a prerequisite of creating complex composite meanings, is yet to be understood. Similarly, whether their concept-label relationships are symbolic in nature or just simple unidirectional associations is not known.

Here we propose a few potential directions for future research that would enable the efficient testing of dogs’ concept manipulation abilities to evaluate the social cognition-mediation theory for language readiness. We argue that using advanced protocols together with exploiting natural biases of dogs can aid in uncovering their potential concept manipulation skills essential for language-readiness.

### Proposed experimental approaches to investigate dogs’ concept manipulation skills

Recent research has shown that neuroscientific experiments using passive paradigms may be more sensitive in revealing certain cognitive abilities or implicit knowledge, which might otherwise remain undetected by currently available performance-based measures (Karuza et al. 2014), and an otherwise potentially existing corresponding behaviour can be obscured by confounding factors inherent to testing conditions. Combining fMRI and EEG measures work from our lab has shown dogs’ ability to human-analogously track regularities when segmenting a speech stream (Boros et al. 2021). A semantic expectation violation ERP paradigm recently adapted for dogs could also successfully demonstrate that dogs possess referential understanding of object-labels (Boros et al. 2024), a capacity previous behavioural experiments using performance measures failed to reveal (Fugazza et al. 2021; Ramos and Mills 2019). We propose that combining the statistical learning paradigms that have proven successful in dogs with sequences that were designed to build up recursive hierarchical structures (Al Roumi et al. 2023; Maheu et al. 2019; Planton and Dehaene 2021) can serve as a suitable test to assess dog brains’ ability to represent information in recursively nested structures. Similarly, semantic expectation violation paradigms can be used to demonstrate symbolic capacities of dogs, because if the learned associations are internalized by them, then reversing the presentation order should not change the magnitude of the violation response as reversibility is an important requisite of symbols (e.g. the word “apple” represents the fruit, and the fruit retrieves the word, van Kerkoerle et al. 2024).

We propose that exploiting natural cross-model correspondences of species can help in revealing previously unreported concept manipulation skills. Specifically, such correspondences may bootstrap label learning in populations that seem to struggle with acquiring large vocabularies. The complex capacity of using symbolic systems may stem from natural biases and associations. Natural cross-modal correspondences (e.g. things that look big give low sounds) lead to spontaneous associations which, in turn, may drive heuristics and expectations on what an individual should see when hearing a certain sound and thus may provide a sufficient underlying mechanism in preverbal humans to enable the establishment of the first links between vocal labels and referents (Imai and Kita 2014). Indeed, humans efficiently make use of these associations, a systematically investigated example of which is referred to as sound symbolism where speech sounds are linked with features of a referent (e.g. rounded vowel-round shape: Maurer et al. 2006, high vowel-small object: Thompson and Estes 2011). Sound-symbolic expectations are formed without any explicit decision in humans (Peiffer-Smadja and Cohen 2019), are robust across cultures (Ćwiek et al. 2022) and even preverbal infants show sensitivity for them (Asano et al. 2015). Importantly, sound-symbolic associations promote word learning (Lockwood et al. 2016). Whether dogs make spontaneous associations between vocal sounds and referents has never been investigated, but they make use of some of the same cross-modal correspondences as humans, associating higher pitch with smaller size (Korzeniowska et al. 2022) and higher pitch with higher spatial position (Korzeniowska et al. 2019; Parise et al. 2014). We suggest that exploring natural sound-symbolic associations and then building labels on these expectations may bootstrap dogs’ label learning for concepts as in humans, because adding a logical link between the label and referent may be a more powerful learning mechanism than simple associations between arbitrary elements. Previous attempts to teach object labels to dogs did not make use of these correspondences, but exploiting sound-symbolic expectations when associating a new label with an object may induce learning of larger vocabularies even in typical dog populations that usually show limited capacity to learn many labels. Additionally, building on labels that represent such sound-symbolic links when testing the reversibility of concept-label association in dogs, may also enhance the probability of detecting their potential symbolic processing abilities.

Another approach that may boost the manifestation of concept manipulations skills is to use concepts that are naturally present in a species’ mind. Evidence suggests that young infants have less stable concepts (e.g. less distinct object categories) (Hadley et al. 2014; Hespos and Spelke 2004; Lewkowicz 2014; Quinn et al. 2016; Sucevic et al. 2017; Xie et al. 2022) and have difficulty deploying them (Piantadosi et al. 2018; Shukla and de Villiers 2021), which may be behind some of their seemingly poorer linguistic abilities (e.g. word learning through mutual exclusivity, access to phonetic details, forming composite meanings). Importantly, however, when the retrieval of conceptual information is induced (e.g. highlighting with pointing, or showing a picture of the object), these complex tasks become solvable even for young, preverbal infants (Mani et al. 2012; Pomiechowska et al. 2021, 2024). Interestingly, efficiently detecting and categorizing event components is also argued to help infants to understand and learn verbs (Golinkoff and Hirsh-Pasek 2008). The nature of conceptual representations may not only show ontogenetic but also species-specific differences, e.g. the stability of categories or where the categorical boundaries are drawn can differ. How dogs naturally categorize continuous experiences have never been investigated but can be revealed by using data-driven approaches to fMRI data analysis (voxel decomposition technique: Norman-Haignere et al. 2015; and RSA: Kriegeskorte et al. 2008). We propose that assigning labels to concepts that are intrinsically present in a species’ mind may bootstrap label learning. Similarly, with inducing pre-existing conceptual knowledge (e.g. making the information salient by highlighting the familiar object with pointing) the complex capacity to form composite meanings may be induced and be detectable in dogs as shown recently in a similar paradigm with preverbal infants (Pomiechowska et al. 2024).

## Conclusions for future biology

To conclude, here we proposed that the emergence of concept manipulation skills essential for language-readiness was mediated by complex social-cognitive skills such as understanding others as intentional agents, attributing goals to them and owning mentalization abilities. We suggested that the domestic dog is a suitable model species to evaluate this social cognition-mediation hypothesis as dogs have been subject to similar selective pressures to those that are likely to have promoted prosociality in early humans. We argued that, as a consequence, dogs show complex social-cognitive skills, and thus, while may not be exclusive in this regard, have a greater potential than other nonhuman animals to develop concept manipulation skills for language-readiness. We have highlighted that by using advanced neural measures, which are by now well-established and reliably applicable in dogs, and by exploiting natural species-specific biases the detectability of species’ concept manipulation skills can be improved. Finding evidence for concept manipulation abilities (i.e. symbolic processing, forming composite meanings, tracking hierarchical nested structures) in dogs would favour that that such abilities can indeed arise as a consequence of possessing complex social-cognitive capacities. On the contrary, finding that dogs lack these key concept manipulation skills would challenge the social cognition-mediation hypothesis and would necessitate finding alternative explanations for what bootstrapped language-readiness in early humans.
